# Supplementary material for: Intergenerational Support and Second-Child Fertility Intention in the Chinese Sandwich Generation: The Parallel Mediation Model of Double Burnout
Source: Behav Sci (Basel). 2023 Mar 14;13(3):256. doi: 10.3390/bs13030256 (PMC10045525; doi:10.3390/bs13030256)
Supplement: Supplementary file 1 [file behavsci-13-00256-s001.zip › behavsci-2212750-supplementary.pdf]

Supporting Information

Table S1. Logistic regression predicting propensity score of supporting.

	Odds Ratio	SE	*p*
Father's socioeconomic status	1.212	0.060	0.000
Mather's socioeconomic status	1.094	0.060	0.000
Father's age	0.974	0.007	0.000
Mather's age	0.919	0.007	0.000
Paternal grandfather's socioeconomic status	0.990	0.006	0.084
Maternal grandfather's socioeconomic status	1.892	0.088	0.000
Paternal grandparents' health condition	0.665	0.087	0.002
Maternal grandparents' health condition	1.026	0.007	0.000
Paternal grandfather's *hukou* (1=rural)	1.487	0.063	0.000
Father's occupational reputation	1.007	0.006	0.094
Mather's occupational reputation	1.006	0.006	0.091
First child's gender (1=men)	1.022	0.087	0.047
Maximum years of parents' education	1.007	0.018	0.707
Living with grandparents or not (1=no)	0.992	0.073	0.012
Firstborn children's age	1.079	0.029	0.005
Constant	0.039	0.017	0.000

Table S2. Propensity score matching covariate balance.

Variable		Mean		% Reduction		t-test	
		Treated	Untreated	%Bias	% Bias Reduction	t	*p*
Father's socioeconomic status	Unmatched	2.513	2.219	32.5	89.1	8.17	0.000
	Matched	2.498	2.428	3.5		0.80	0.422
Mather's socioeconomic status	Unmatched	2.420	2.202	31.5	88.2	8.17	0.000
	Matched	2.361	2.364	3.1		0.79	0.419
Father's age	Unmatched	37.096	37.724	-15.8	86.4	-4.01	0.000
	Matched	36.794	37.742	-2.1		-0.47	0.640
Mather's age	Unmatched	36.266	36.315	-11.8	85.9	-4.11	0.000
	Matched	36.882	36.625	-2.5		-0.54	0.619
Paternal grandfather's socioeconomic status	Unmatched	33.751	32811	8.9	77.2	2.26	0.024
	Matched	33.748	33.736	-2.0		-0.49	0.623
Maternal grandfather's	Unmatched	32.124	31.241	10.0	86.5	2.49	0.013

socioeconomic status							
	Matched	32.121	32.132	-1.3		-0.33	0.90
Paternal grandparents' health condition	Unmatched	4.019	3.388	67.4	94.3	17.06	0.000
	Matched	3.998	3.879	3.8		0.86	0.389
Maternal grandparents' health condition	Unmatched	3.906	3.426	46.6	88.6	11.98	0.000
	M atched	3.876	3.895	5.3		1.15	0.249
Paternal grandfather's *hukou* (1=rural)	Unmatched	1.213	1.221	-8.6	84.9	-2.21	0.027
	Matched	1.179	1.184	1.3		0.28	0.782
Father's occupational reputation	Unmatched	40.778	39.351	15.7	93.7	3.97	0.000
	Matched	40.742	40.753	1.0		0.23	0.815
Mather's occupational reputation	Unmatched	39.639	38.157	15.2	93.5	3.94	0.000
	Matched	39.731	40.766	1.0		0.23	0.817
First child's gender (1=men)	Unmatched	1.483	1.459	-9.62	85.4	-0.17	0.000
	Matched	1.485	1.475	1.2		0.36	0.449
Living with grandparents or not (1=no)	Unmatched	1.305	1.096	-2.9	81.3	-0.74	0.461
	Matched	1.301	1.236	0.5		0.11	0.910
Firstborn children's age	Unmatched	9.871	7.112	56.2	96.5	3.45	0.025
	Matched	9.195	8.964	3.8		0.44	0.96

Sample	Pseudo-R^2	χ^2	p>chi^2	MeanBias	MedBias	B	R	%Var
Unmatched	0.117	425.49	0.000	18.3	10.1	86.4*	1.04*	64
Matched	0.002	5.31	0.724	2.2	1.8	10.5	0.71	27

Note. p values are from the t-tests(two-tailed). * if B>25%, R outside [0.5; 2].

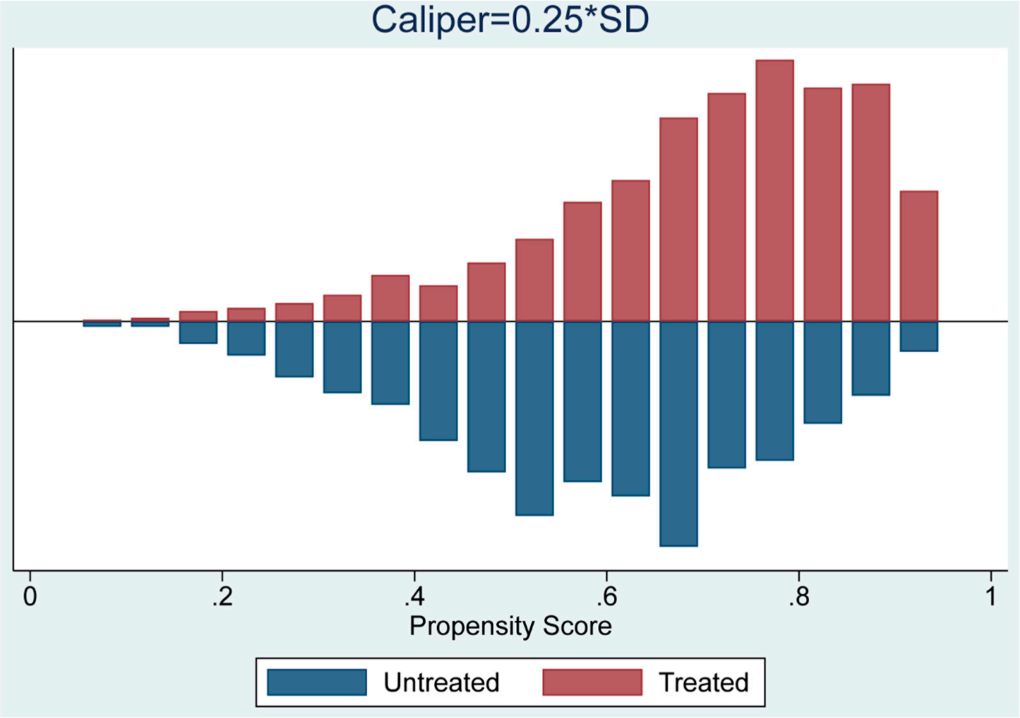

Figure S1. Common support of treated and untreated sample.

Table S3. Average treatment effect (ATT) of IS on PB, BCG, and SCFI in Chinese sandwich generation.

		PB	BCG	SCFI
		ATT	ATT	ATT
Nearest neighbor Matching	Difference	-3.201***	.289*	.532***
	T-stat	-3.77(.830)	0.52(.661)	8.14 (.051)
Radius Matching	Difference	-2.306***	.090	.614***
	T-stat	-5.12(.425)	0.43(.325)	15.98(.035)
Kernel matching	Difference	-2.011***	.012	.560***
	T-stat	-2.73(.601)	-0.20(.623)	11.51(.048)
Local Linear Matching	Difference	-2.284***	.108	.517***
	T-stat	-2.97(.667)	0.11(.61)	10.13(.049)
Stratification and Interval	Difference	-2.007***	-.105	.539***
Matching	T-stat	-3.08(.543)	0.11(.051)	10.672(.0.099)
Mahalanobis distance matching	Difference	-3.085***	.197*	.639***
	T-stat	-1.043(0.293)	0.161(0.081)	9.27(0.182)

Note. * $p < 0.05$ ** $p < 0.01$ *** $p < 0.001$.

Table S4. Results of sensitivity analysis via regressions using different calipers in PSM.

Gamma	Sig+	Sig-	t-hat+	t-hat-	CI+	CI-
1	0	0	0.53	0.86	0.566	0.598
2	8.7E-15	0	0.493	0.848	0.575	0.872
2.1	3.6E-12	0	0.491	0.856	0.482	0.897
2.2	6.1E-10	0	0.409	0.825	0.432	0.865
2.3	4.6E-08	0	0.442	0.901	0.398	0.906

2.4	1.8E-06	0	0.401	0.811	0.271	0.932
2.5	0.000011	0	0.391	0.868	0.220	0.945
2.6	0.000024	0	0.366	0.854	0.239	0.958
2.7	0.00031	0	0.353	0.878	0.211	0.975
2.8	0.0039	0	0.391	0.911	0.209	0.965
2.9	0.0041	0	0.305	0.998	0.102	0.996
3	0.0057	0	0.296	0.938	0.065	1.001
3.1	0.05127	0	0.263	0.962	0.027	1.062
3.2	0.129995	0	0.241	0.992	0.016	1.084
3.3	0.25831	0	0.198	1.102	0.002	1.154
3.4	0.56321	0	0.143	1.166	-0.014	1.254
3.5	0.87245	0	0.122	1.257	-0.029	1.268
5	1	0	-0.101	1.384	-0.124	1.293
8	1	0	-0.332	1.427	-0.166	1.296
10	1	0	-0.467	1.562	-0.185	1.311

Note: gamma - log odds of differential assignment due to unobserved factors; sig+ - upper bound significance level; sig- - lower bound significance level; t-hat+ - upper bound Hodges-Lehmann point estimate; t-hat- - lower bound Hodges-Lehmann point estimate; CI+ - upper bound confidence interval (a=.95); CI- - lower bound confidence interval (a=.95).
